# Identifying Risk Factors for Complications During Exposure for Anterior Lumbar Interbody Fusion

**DOI:** 10.7759/cureus.16792

**Published:** 2021-07-31

**Authors:** William G Wert, William Sellers, David Mariner, Melissa Obmann, Boyoung Song, Evan J Ryer, Shivprasad Nikam

**Affiliations:** 1 General Surgery, Geisinger Wyoming Valley Medical Center, Wilkes-Barre, USA; 2 Vascular Surgery, Geisinger Wyoming Valley Medical Center, Wilkes-Barre, USA; 3 Endovascular and Vascular Surgery, Geisinger Medical Center, Danville, USA

**Keywords:** spine surgery, vascular complication, venous injury, vascular surgeon, alif, anterior lumbar interbody fusion

## Abstract

Introduction

An anterior approach to lumbar interbody fusion is a widely utilized method of access to the lumbar spine. Due to the potential for vascular complications with spine exposure, vascular surgeons are frequently included in the care of these patients as part of a team-based approach. Identifying risk factors for such complications is difficult and not well-defined in the literature. In this investigation, we evaluate the potential risk factors for complications during anterior lumbar inter-body fusion (ALIF).

Methods

This is a retrospective review of 106 patients who underwent ALIF at a single institution between May 1, 2007, and April 30, 2017. Patients were identified through operating room case logs and Current Procedural Terminology (CPT) codes correlating with ALIF. Vascular surgeons performed all anterior exposures. Patient demographics and data regarding their surgical care and postoperative course were obtained from a review of operative and progress notes in the electronic medical record. Statistical methods employed included a t-test for normally distributed data and the Wilcoxon rank-sum test for non-normally distributed data. Categorical variables were compared using Fisher’s exact and chi-square tests. A logistic regression model was applied to predict complications by controlling other significant covariates.

Results

Of the 106 patients included in this analysis, 16 patients experienced a defined complication, giving an overall complication rate of 15%. Patients with complications were more likely to be of male gender (n=11, P=0.016), with older average age (54.6, P=0.017), with higher estimated blood loss, with higher use of blood products, and with higher use of cell-saver. A venous injury was the most common complication (n=11, 10.4%); ileus and nerve injury were the next most common (n=3, 2.8%). The 30-day mortality was 0%. Male gender demonstrated an odds ratio of 3.78 (P=0 .034) in a logistic regression model after adjusting for age and blood products.

Conclusions

Overall complication rates were comparable to those in the published literature and male gender was identified as a predictor for risk of complications in those undergoing ALIF. This is the first study to identify male sex as a risk factor for complications following ALIF. The results of this study will hopefully guide future studies in gaining more insight into the predictors of complications in larger series.

## Introduction

First described by Ito for the treatment of Pott’s disease in 1934 [1], anterior lumbar interbody fusion has grown so popular that almost 100,000 such operations have been performed over the past decade [2]. Today, indications for lumbar fusion include degenerative disc disease, scoliosis, spondylolisthesis, adjacent segment disease, and spinal instability. Several approaches to lumbar fusion have been developed over the years, including anterior, posterior, combined anterior-posterior, lateral, axial, transforaminal, laparoscopic, and robot-assisted techniques [2-3]. Each approach brings certain advantages, risks, and complications. Anterior lumbar interbody fusions (ALIF) are considered advantageous over posterior approaches since they do not require dissection or retraction of the posterior spinal musculature and allow for improved visibility of the anterior column and disc space. Conceptual benefits of ALIFs include the restoration of disc height and lumbar lordosis, reduction of anterolisthesis, achievement of coronal and sagittal balance, decreased adjacent segment disease, and fewer injuries to the dura and posterior neural structures [3-4]. Due to the risk of vascular injury and the need for mobilization of major vasculature, ALIF typically requires a multidisciplinary approach. Orthopedic surgeons or neurosurgeons often work in tandem with general and vascular surgeons to gain exposure of the spine [4-6].

The complication rate of ALIF has been reported to be as high as 40% with mortality rates ranging from zero to four percent [2-21]. Risks include skin and spinal infection, wound dehiscence, hernia, seroma, ileus, bleeding, thrombosis/embolism, and injury to bowel, nervous and genitourinary structures [4,7]. Vascular injury is generally the most feared complication, and the incidence is reported to be as high as 15.6% [6,8-11].

Many peri-operative characteristics have been identified in previous studies as being risk factors for complications. These include multilevel spinal exposure, surgeon inexperience, age, obesity, and prior spinal surgery, however, reproducibility of these findings has been limited [12-14]. There also seems to be no firm consensus in the literature about which spinal level intervention results in the most complications [12,15]. The aim of this study is to examine the complications of ALIFs and identify the risk factors that predispose to these complications. We hypothesize that vascular injury would be the most common complication resulting from ALIF, and that patient characteristics contributing to the difficulty of exposure such as obesity and previous abdominal surgeries as well as older age with higher numbers of comorbidities would be significant risks factors for such complications.

## Materials and methods

A retrospective review of 106 patients who underwent ALIF at a single institution from May 1, 2007, to April 30, 2017, was conducted. Approval for this study was granted after review by our health system’s Institutional Review Board (IRB). Since this was a retrospective chart review, informed consent was not required. Inclusion criteria for this study were adults over age 18 who underwent ALIF. Exclusion criteria included age less than 18 years, pregnancy, and those patients with incomplete medical records or those who were lost to follow-up. Patients were identified from the electronic medical record (EMR) using the International Classification of Diseases, Ninth Revision (ICD-9) and Current Procedural Terminology (CPT) codes. Demographics, clinical characteristics, and radiologic and operative data were obtained from the EMR. Perioperative complications for all patients were examined up to 30 days postoperatively and included arterial injury, venous injury, prolonged postoperative ileus, impotence, retrograde ejaculation, bowel injury, genitourinary injury, nerve injury, erectile dysfunction, venous thromboembolism, surgical site infection, cerebrovascular accident, and 30-day mortality. 

All patients underwent exposure of the lumbar spine by a board-certified vascular surgeon utilizing a retroperitoneal approach. Surgical incisions were either a low transverse or a paramedian incision of the rectus sheath. Vascular anatomy and the number of levels requiring exposure determined the choice of incision. Continuous variables were compared using the t-test for normally distributed data and the Wilcoxon rank-sum test for non-normally distributed data. Categorical variables were compared using Fisher’s exact and chi-square tests. A logistic regression model was fit to predict the patients who have complications or not by controlling other significant covariates. All data analyses were performed using SAS software version 9.4 (SAS Institute Inc., Cary, NC).

## Results

A total of 106 patients met inclusion and exclusion criteria and were entered into this analysis. Of the 106 patients, 90 (85%) were without complications and 16 (15%) experienced a complication associated with ALIF. The mean age of the study population was 47.5 years, and 44 patients (42%) were male. The average body mass index (BMI) of patients included in the study was 29.5 kg/m^2^ with an average height of 1.7 meters and an average weight of 85.4 kg. Further details regarding our patient’s demographics can be found in Table 1. Potential risk factors and comorbidities included current tobacco use status in 53 patients (49%), any tobacco use history in 77 patients (73%), chronic kidney disease (n=1, 1%), diabetes mellitus (n=6, 6%), hypertension (n=33, 31%), coronary artery disease (n=7, 7%), chronic obstructive pulmonary disease (n=7, 7%), history of venous thromboembolism (n=4, 4%), and previous malignancy (n=5, 5%). Sixty-three patients (59%) had one or more previous abdominal surgeries. A single patient (1%) had a previous anterior spinal surgery and 53 patients (50%) had previous posterior spinal surgery. Further details regarding risk factors and comorbidities are outlined in Table 2. The median estimated blood loss was 150 ml, and 36 patients (34%) required the administration of blood products of some form. Six patients (6%) required transfusion of packed red blood cells and 34 patients (32%) required autologous transfusion with the cell-saver. Three patients (3%) required transfusion of fresh-frozen plasma. Blood loss and blood products used are outlined in Table 3. A single spinal-level procedure was performed on 45 patients (43%), two spinal levels on 43 patients (41%), three spinal levels on 16 patients (15%), and four spinal levels on two patients (2%). L3-L4 was operated on in seven patients (7%), L4-L5 in 52 patients (49%), L5-S1 in 91 patients (86%). Details regarding specific spinal levels and the number of spinal levels operated on are outlined in Table 4.

**Table 1:** Clinical characteristics of 106 patients undergoing exposure for anterior lumbar interbody fusion (ALIF) by vascular surgeons BMI: body mass index; *Standard Deviation

Characteristic	All patients (n=106)	Without complication (n=90)	With complication (n=16)	P-value
Male gender (%)	44 (41.5%)	33 (36.7%)	11 (68.5%)	0.0164
Age, mean (STD*)	47.5 (13.2)	46.2 (12.2)	54.6 (16.4)	0.0173
BMI, mean (STD*)	29.5 (5.8)	29.6 (5.7)	29.1 (6.4)	0.7230
Height (m), mean (STD*)	1.7 (0.1)	1.7 (0.1)	1.7 (0.1)	0.5976
Weight (kg), mean (STD*)	85.4 (20.2)	85.5 (20.2)	84.9 (20.9)	0.9103
Current tobacco use	52 (49.1%)	45 (50.0%)	7 (43.8%)	0.6449
Any tobacco use	77 (72.6%)	65 (72.2%)	12 (75.0%)	0.9999

**Table 2:** Comorbidities of patients undergoing exposure for anterior lumbar interbody fusion (ALIF) by vascular surgeons

Comorbidity	All patients (n=106)	Without Complication (n=90)	With Complication (n=16)	P-value
Prior abdominal surgery	63 (59.4%)	57 (63.3%)	6 (37.5%)	0.0525
Prior anterior spinal surgery	1 (0.9%)	1 (1.1%)	0 (0%)	0.9999
Prior posterior spinal surgery	53 (50.0%)	42 (46.7%)	11 (68.8%)	0.1035
Chronic kidney disease	1 (0.9%)	0 (0%)	1 (6.3%)	0.1509
Diabetes mellitus	6 (5.7%)	4 (4.4%)	2 (12.5%)	0.2228
Hypertension	33 (31.1%)	26 (28.9%)	7 (43.8%)	0.2527
Congestive heart failure	0 (0%)	0 (0%)	0 (0%)	n/a
Coronary artery disease	7 (6.6%)	4 (4.4%)	3 (18.8%)	0.0682
Chronic obstructive pulmonary disease	7 (6.6%)	4 (4.4%)	3 (18.8%)	0.0682
Venous thromboembolism	4 (3.8%)	3 (3.3%)	1 (6.3%)	0.4856
Malignancy	5 (4.7%)	5 (5.6%)	0 (0%)	0.9999

**Table 3:** Perioperative blood loss and use of blood products in patients undergoing exposure for anterior lumbar interbody fusion (ALIF) by vascular surgeons

Blood product	All patients (n=106)	Without complication (n=90)	With complication (n=16)	P-value
Estimated blood loss (ml), median (IQR)	150 (100, 270)	150 (100, 250)	400 (200, 575)	0.0005
Blood products	36 (34.0%)	26 (28.9%)	10 (62.5%)	0.0089
Packed red blood cells	6 (5.7%)	4 (4.4%)	2 (12.5%)	0.2228
Cell saver	34 (32.1%)	24 (26.7%)	10 (62.5%)	0.0047
Fresh frozen plasma	3 (2.8%)	3 (3.3%)	0 (0%)	0.9999

**Table 4:** Comparison of complications by the number of spinal levels exposed in patients undergoing anterior lumbar interbody fusion (ALIF) by vascular surgeons

Spinal levels	All patients (n=106)	Without complication (n=90)	With complication (n=16)	P-value
				0.0223
1	45 (42.5%)	43 (47.8%)	2 (12.5%)	
2	43 (40.6%)	34 (37.8%)	9 (56.3%)	
3	16 (15.1%)	11 (12.2%)	5 (31.3%)	
4	2 (1.9%)	2 (2.2%)	0 (0%)	

When comparing the characteristics of the patients with complications to those without, we found that there were significantly more male patients in the group that experienced a complication with ALIF (69% vs. 37%, P=0.016). Furthermore, the mean age of those patients with a complication was significantly greater than those without (55 years vs. 46 years, P=0.017). Moreover, the estimated blood loss in patients with complications was significantly higher than those without a complication (400 ml vs. 150 ml, P=0.0005). Correspondingly, blood product use was higher in patients with a complication (63% vs 29%, P=0.009) as was the rate of cell saver use (63% vs 27%, P=0.005). In contrast, the average body mass index (BMI) of patients with complications was not significantly different between the two comparison groups (29.1 kg/m^2^ vs. 29.6 kg/m^2^, P=0.72). Contrary to our hypothesis, we found that a history of previous abdominal surgeries or the number of previous abdominal surgeries did not show a statistically significant difference between patients with and without complications in our study population. Patients without complications, in fact, were found to be more likely to have undergone previous abdominal surgeries (n=57, 63%) when compared to those patients with complications (n=6, 38%); however, this comparison failed to reach statistical significance (P=0.053). Additionally, there was no statistically significant difference in the rate of complications in patients who suffered from any of the comorbidities outlined in Table 2 or with reference to the specific spinal level operated upon. There was, however, a significant difference in complications when evaluating the number of spinal levels operated on (P=0.022). Table 4 shows two-level spinal surgery carrying the highest risk of complications at 56% (n=9). A history of previous anterior and posterior spinal surgery was not found to be significantly different between those with and those without complications.

The most common complication encountered in our cohort was venous injury and this was found in 11 patients (10%). Other less common complications include postoperative ileus in three patients (3%), impotence and retrograde ejaculation were each seen in one patient (1%), nerve injury in three patients (3%), and venous thromboembolism in two patients (2%). In our series, there were no instances of arterial injury/thrombosis, bowel injury, genitourinary injury, surgical site infection, or cerebrovascular accidents. Furthermore, our 30-day mortality was zero percent. The complications encountered in our study are summarized in Table 5. In an effort to identify potential risk factors for complications following ALIF, we performed a logistic regression analysis for predictors of complications. After adjusting age and the use of blood products, only male gender proved to be a significant predictor of perioperative complications, with an odds ratio of 3.738 (CI 1.104-12.653, P=0.0341). Further details of our logistic regression analysis can be found in Table 6.

**Table 5:** Perioperative morbidities incurred by patients undergoing exposure for anterior lumbar interbody fusion (ALIF) by vascular surgeons

Complication	n (%)
Arterial injury	0 (0%)
Venous injury	11 (10.4%)
Ileus	3 (2.8%)
Impotence	1 (0.9%)
Retrograde ejaculation	1 (0.9%)
Bowel injury	0 (0%)
Genitourinary injury	0 (0%)
Nerve injury	3 (2.8%)
Erectile dysfunction	1 (0.9%)
Venous thromboembolism	2 (1.9%)
Wound infection	0 (0%)
Cerebrovascular accident	0 (0%)
30-day mortality	0 (0%)

**Table 6:** Logistic regression analysis evaluating risk factors for complications in patients undergoing exposure for anterior lumbar interbody fusion (ALIF) by vascular surgeons

Characteristic	Odds ratio	95% CI		P-value
Gender (male)	3.738	1.104	12.653	0.0341
Age	1.039	0.997	1.082	0.0704
Blood products	4.168	1.276	13.610	0.0181

## Discussion

In our present series of patients undergoing ALIF over a 10-year period, with exposure performed by a vascular surgeon, the percentage of patients with at least one complication was 15% and compares favorably to the current literature. Of late, there have been a number of articles published comparing the complication rates of spinal surgeons versus access teams that consist of a vascular or general surgeon in tandem with the spinal surgeon to obtain exposure, and these studies have provided mixed results. In 2009, Jarrett, et al. reported an 8% overall complication rate for spinal surgeons compared to a 12% complications rate with a team approach [16]. In contrast, Qurashi et al. in 2012 reported an overall complication rate of 20% when performed by spinal surgeons alone [17]. Certainly, a thorough knowledge of the surrounding anatomy is paramount in performing any successful surgical procedure, and the ability to recognize and correct both major and minor complications intraoperatively may be the difference between a successful procedure and major morbidity. As in many institutions, we have gravitated toward a team approach to reduce complications and provide a more personalized and presumably safer approach to each individual spine patient.

In our study, venous injury accounted for the majority of the complications encountered. While we did not make the determination between major and minor vascular injuries, specify which vessel was injured, or the approach to repair, our overall incidence of vascular injury was 10.4%. This is similar to most but slightly higher than some of the series in the reported literature. For example, Smith et al. reported a 3.6% incidence of vascular injury [18] while others report similar results of 11% such as Hamdan et al., in a study of 480 patients undergoing ALIF [8]. Chiriano et al. reported a vascular injury rate of 26% [15]. Certainly, any vascular injury has the potential for major morbidity when operating in this field. The iliac vessels and perforating lumbar vessels typically encountered during this procedure can bleed profusely. Even once repaired, this type of injury may still have a predilection for future morbidity. A study performed by Nourian et al. in 2015 performed a retrospective review of 204 patients undergoing ALIF and found that those who sustained a vascular injury intraoperatively were much more likely to develop deep venous thrombosis than those who did not, 36% and 5%, respectively [19].

Interestingly, we did not find elevated BMI to be related to higher complications in our study population. Theoretically, the increased depth of tissue, decreased operative domain and a more challenging exposure could lead to increased vascular complications in obese patients, but our findings did not reflect this. A study conducted by Peng et al. in 2009 evaluated this as well and found obese and non-obese patients to be comparable in terms of complications [20]. This is in contrast to other studies that do show an increased risk of complications in patients with a BMI greater than 30 kg/m^2^ [12-14]. Looking at our patient demographics, one may draw the conclusion that the average BMI of our study population was 29.5 kg/m^2^ and that obese patients were, therefore, underrepresented; however, the average BMI of our patient population was comparable to other studies [12-14,20]. 

 The results of our initial analysis show a statistically significant difference in age for those with and without complications from ALIF, with complications more likely in older patients. However, when we performed our logistic regression model and adjusted for the sex and blood loss, the odds ratio of complications in older age patients was only 1.039, with an insignificant P-value of 0.0704. This is contradictory to our hypothesis that older patients may have higher complication rates presumably due to comorbidities, history of previous surgery, and less compliant blood vessels. A study performed by Rothenfluh et al. in 2013 evaluated complications in ALIF patients over the age of 60 and found that these patients do not have higher overall complication rates than their younger cohort [21]. Ballard et al. (2014), however, found that age over 69 years was associated with an increased rate of wound hematoma [13].

We found statistical significance in the incidence of complications associated with the number of spinal levels operated on during ALIF, with two consecutive levels having the highest incidence. Nine patients (56%) of those with complications in our study underwent two-level ALIF. We did not find any statistical significance, however, based on individual spinal levels. Hamdan et al. studied this and did find a difference, with L4-L5 carrying the highest risk of complications with 83% of complications occurring in ALIFs at this level [8]. The larger size of this study, evaluating 480 patients compared to our 106, may well account for the discrepancy. Estimated blood loss (EBL) was understandably higher and therefore associated with complications in our study population. The majority of our complications were vascular injuries, and it should be expected that those who suffer vascular injury will have a higher EBL and increased need for transfusion and/or a cell saver. Of all the risk factors that we evaluated, male gender was found to be most predictive of complications in our study population. Though our study population included more women than men (58% vs 42%), after adjusting age and blood products used in our logistic regression, the odds of any complication were 3.738 times in male patients with a P-value of 0.0341. This is contradictory to other studies, which do not show any significant gender-related predisposition to complications. With a further evaluation of this data in our logistic regression model with an r-square analysis, we get an r-square value of 0.1352 and a max-rescaled r-square of 0.2363. The c-statistics of this is 0.786 with values over 0.7 indicating a good model.

 Our study was retrospective in nature and thus limited by the data available and the analysis that could be performed. The study size is relatively smaller than some larger studies in the literature. Though limited, this study was still able to provide useful information, identifying the male gender as being at increased risk for complications during ALIF. Though the c-statistics of our logistic regression of 0.786 may suggest a good model, the r-square is inarguably low. We only found three variables to include in our logistic regression, which likely resulted in this low number. With a larger study, we may be able to identify more risk factors that would generate a better model for prediction.

## Conclusions

In conclusion, we found in our retrospective study of 106 patients undergoing ALIF at a single institution that venous injury was the most common complication in our study population at a rate of 10%, and the overall rate of complications was 15%. Other factors, such as age, obesity, and prior abdominal surgery, were not shown to predict a significant increase in risk for complications as suggested in other studies. Furthermore, we are the first study to show the male gender demonstrating an increased risk with an odds ratio of 3.7 for complications from this procedure. Future work will need to be done on this topic and further studies will be needed to identify other unknown risk factors for perioperative complications in patients undergoing vascular exposure for anterior lumbar interbody fusion.
